# Extensive ICP-MS and HPLC-QQQ detections reveal the content characteristics of main metallic elements and polyphenols in the representative commercial tea on the market

**DOI:** 10.3389/fnut.2024.1450348

**Published:** 2024-08-12

**Authors:** Yanlin An, Dahe Qiao, Tingting Jing, Shize Li

**Affiliations:** ^1^Department of Food Science and Engineering, Moutai Institute, Renhuai, China; ^2^Tea Research Institute, Guizhou Academy of Agricultural Sciences, Guiyang, China; ^3^State Key Laboratory of Tea Plant Biology and Utilization, Anhui Agricultural University, Hefei, China; ^4^College of Life Sciences, Guizhou University, Guiyang, China

**Keywords:** six categories of tea, metal element content, polyphenols, limited standards, consumer choice

## Abstract

The content of polyphenols and metal elements in tea has an important impact on the choice of consumers. In this study, we conducted a comparative analysis of ten elements including Fe, Mg, Al, Zn, Cu, Mn, Ni, Cr, Pb, and As in 122 representative tea samples from 20 provinces. The results showed that the difference of metal content among six tea categories was greater than that among provinces, and the overall metal content of black tea was relatively higher. The contents of all elements from high to low were: Mg > Mn > Al > Fe > Zn > Cu > Ni > Cr > Pb > As. The contents of Ni, Fe, Al, Zn and Mn showed significant differences among multiple types of tea categories. While the detection rates of Pb and As were 10.7 and 24.6%, respectively. The contents of all elements were in line with the national limit standards. Meanwhile, the relative contents of theanine, caffeine and a total of 53 polyphenolic compounds in 122 tea samples were detected. The analysis showed that the content of these compounds differed least between green and yellow tea, and the largest difference between black tea and oolong tea. This study provides important support for consumers to choose tea rationally.

## 1 Introduction

The tea plant (*Camellia sinensis*) originated in ancient China more than 2,000 years ago. Now, it has been widely cultivated in many countries around the world and has become an important economic crop (1). According to different processing techniques, the tender leaves of the tea plant can be processed into green tea, black tea, white tea, dark tea, yellow tea and oolong tea (2). These teas not only have pleasant aroma components, but also are rich in various healthy and beneficial components such as flavonoids, polyphenols, alkaloids, and amino acids (3, 4). Therefore, tea drinking has become one of the most popular non-alcoholic green beverages (5). China was rich in tea resources, with more than 300 varieties and thousands of germplasm resources. There were also at least hundreds of common tea brands sold on the market (6). These teas are not only purely different in origin and processing technology, but also have different quality and characteristic components. Meanwhile, in recent years, there have been frequent cases of excessive heavy metals in some agricultural products, which has caused confusion and health concerns for ordinary consumers when making a choice (7–9).

In addition to characteristic taste substances such as catechins, caffeine and theanine, tea is also rich in trace metal elements such as Fe, Zn, Se and Al. However, with the continuous use of fertilizers and pesticides and their accumulation in the soil, some heavy metal elements can also enter the tea, thereby posing a threat to human health (10, 11). Previous studies have demonstrated that the pH value of the soil may influence the contents of certain metal elements in the leaves of the tea plant, and there is a risk that Pb exceeds the limit value (12). Also, the different processing techniques can have an impact on the heavy metal content in tea (13); Zhang et al. (14) through the research on the soil and tea in Guizhou tea gardens showed that drinking the tea soup brewed with young tea leaves does not pose potential health risks to adults. However, the risks to adults from drinking the tea soup brewed with mature tea leaves are mainly contributed by Mn and Al. Zhong’s detection results of the lead, cadmium, chromium, copper and nickel contents in 25 tea samples from China also imply the risk of heavy metal exceeding the standard (10). These findings all increase consumers’ anxiety when selecting commercially available tea. Currently, there is still a lack of further large-scale comparative analysis of the metal content among different origins or tea categories.

Tea is rich in bioactive substances such as polyphenols, flavonoids, alkaloids and amino acids, among which, polyphenols including catechins and caffeine are considered to be important sources of bitterness and astringency in tea, so they have also become an important basis for consumers to judge the quality of tea (15–17). Previous studies have shown that characteristic metabolites such as catechins and caffeine in tea can change significantly with leaf development, seasonal changes, different processing techniques and varieties, and excessive intake of these substances may cause liver damage, anxiety, insomnia and addiction (European Food Safety Agency, EFSA) (18–22). Zhuang et al. (23) evaluated the polyphenol content in 47 green tea samples and its relationship with astringency. However, this result was lack of research on black tea, oolong tea, white tea, dark tea and yellow tea, which needs to be further widely evaluated. For ordinary consumers, it is still difficult to choose a tea that suits their preferred flavor. In addition, caffeine and theanine not only have important health benefits, but also contribute bitterness and fresh flavor in tea, respectively. And their content variation characteristics need to be revealed on a wider scale (24, 25).

In this study, 122 tea samples from 20 provinces containing six major tea categories were collected. The contents of ten elements such as Fe, Mg, Al, Cu, Zn, Cr, Pb, As, Ni, and Mn in these samples were detected by ICP-MS. Meanwhile, comprehensive detection, comparison, and analysis were conducted on representative flavor substances such as polyphenols, caffeine, and theanine in these samples. Through multiple statistical analyses, our results reveal the main differences in the contents of metals and polyphenols in representative tea samples of different tea categories and regions. The information provides more comprehensive data support for consumers to understand the quality characteristics of commercially available tea.

## 2 Materials and methods

### 2.1 Sample collection

**Figure 1:**
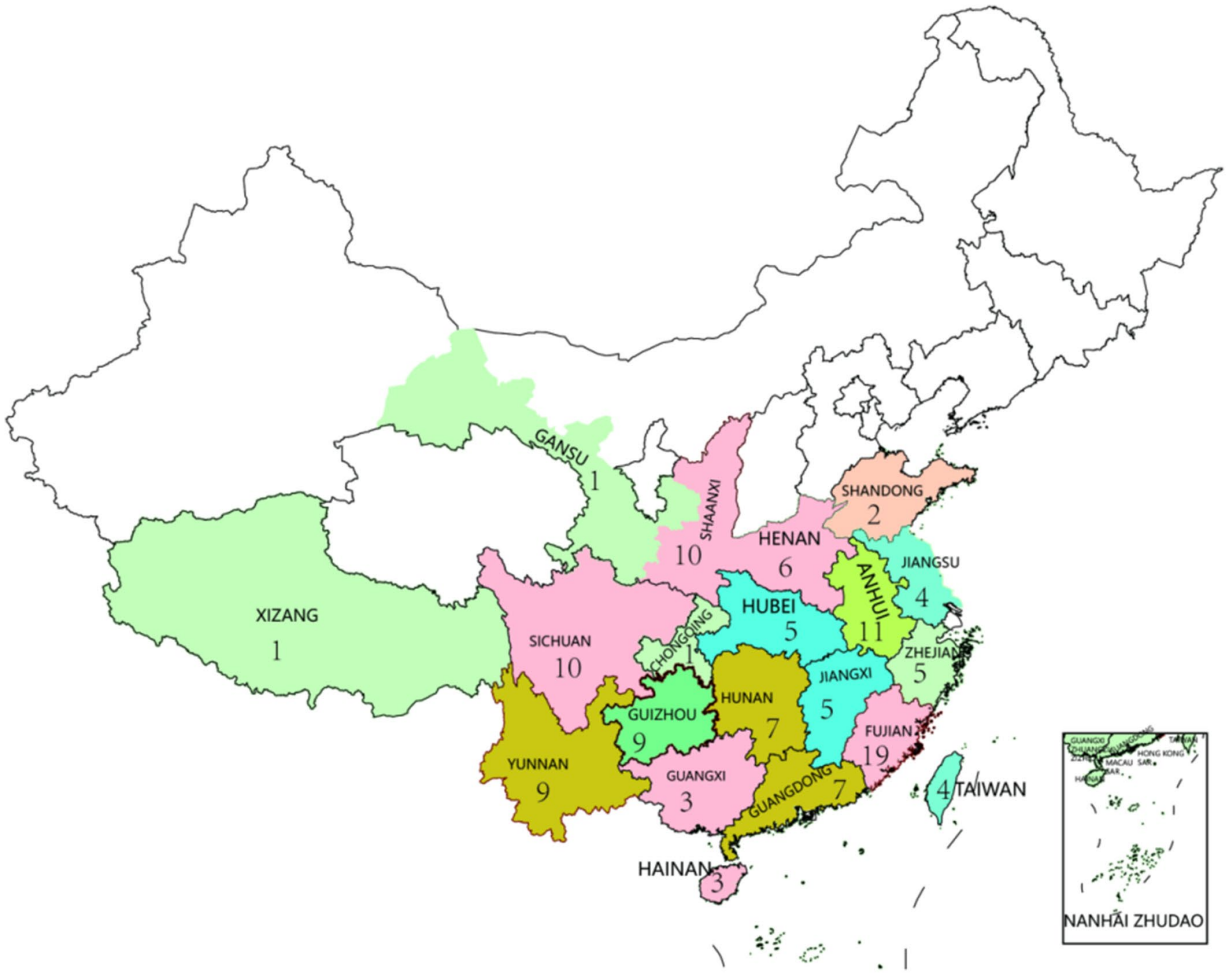
The regional distribution map of 122 tea samples.

A total of 122 tea samples from 20 provinces, including six major tea categories, were collected from the market (Figure 1). Only one sample from Chongqing, Xizan and Gansu was collected, while more than eight samples were collected from the main tea-producing areas of Yunnan, Guizhou, Anhui, Shanxi and Sichuan, and 19 samples from Fujian Province. Among these samples, there are 71 green tea, 17 black tea, 8 dark tea, 15 oolong tea, 6 yellow tea and 5 white tea samples.

### 2.2 Sample pretreatment and metal and potentially toxic element detection

After the samples were collected from the market, they were crushed with a grinder and passed through a 60-mesh sieve, and then stored in a refrigerator at-20°C for later use. A total of 0.300 g of tea samples were placed in quartz digestion tubes with 2.00 mL of ultrapure water and 3.00 mL of nitric acid and self-digested in a fume hood for 12 h. Add 150.00 mL of water and 5.00 mL of nitric acid to the outer tank of the digester and mix well. Subsequently, the self-digestion sample is placed in a microwave digester and microwaved digestion was performed according to standard operating guidelines. The specific steps were detailed in Table 1. After the digestion was completed, the digestion tube was opened and placed on the acid evaporation electric heating plate. The acid was evaporated at 120°C for 2 h. Finally, it was made up to 50.00 mL with ultrapure water for standby. Each sample has three replicates. All the samples were diluted and then injected for detection. The average value of three repetitions was regarded as the final metal element content.

**Table 1:** Microwave digestion step.

Step	Maximum power (W)	Temperature (°C)	Pressure (kPa)	Maintain time (min)
1	1,500	120	15,000	8
2	1,500	220	15,000	12
3	1,500	220	15,000	10

The standard solution was made up to 50.00 milliliters with 0.5% nitric acid water. The standard curve concentrations of different elements are as follows: Cu, Zn, Ni: 0, 0.5, 1.0, 5.0, 10.0, 25.0 and 50.0 ng/mL; Cr, Pb and As: 0, 0.05, 0.1, 0.5, 1, 2.5 and 5 ng/mL; Mn, Mg, Al and Fe: 0, 0.05, 0.1, 0.25, 0.5, 1.0 and 3.0 μg/mL. According to the instrument conditions, the prepared multi-element working solutions were injected and analyzed successively. Taking the injection concentration of each element as the abscissa and the element count response value measured by the instrument as the ordinate, a standard curve was drawn, and the linear regression equation and determination coefficient (R2) were calculated.

Before the formal detection of the sample, the determination conditions and stability of ICP-MS was set and optimized first (26). The pressure of the vacuum system was <6.0 × 10^−8^ kPa, the argon pressure was 0.60 ~ 0.70 MPa. After starting the machine and igniting, a 1 ng/mL tuning solution was used for tuning. The atomizer pressure is 150 ~ 250 kPa, the ICP forward power was 1,200 ~ 1,300 W, the plasma gas flow rate was 14.0 ~ 15.0 L/min, and the atomizer flow rate was 0.8 ~ 1.2 L/min, so that the instrument reaches a stable and accurate state. Then, the pipeline was cleaned with 1.0% nitric acid water for 20 min. The common reaction cell analysis mode was selected for the detection method, the collection mode was peak skipping, the scanning times are 100 times, and the data collection was repeated 3 times. The above methods mainly refer to the national standard GB 5009.268 (27). In the above national standards, the detection limit and quantification limit of each element have been listed in detail.

### 2.3 Detection of polyphenols content

For the determination of polyphenols, the following is the sample preparation method: In the first step, 0.0306 g of tea was weighed and placed in a 2 mL centrifuge tube; in the second step, 1 mL of 80% methanol solution was mixed with the sample and sonicated for 20 min at 4°C; and in the third step, the sample was centrifuged at 10,000 rpm and 4°C for 10 min, and the supernatant was transferred into a new 2 mL centrifuge tube; and in the fourth step, 1 mL of 80% methanol solution was piped, and the supernatant was combined after repeating steps 2 and 3. The extracts were filtered with 0.22 μm aqueous membrane before mass spectrometry. An AB sciex triple quad 5,500+ (Shimadzu Corporation) system was used for polyphenols analysis. The compounds are relatively quantitatively detected by using the MRM mode of the HPLC-QQQ-MS/MS system. The specification of the chromatographic column was: ODS-3 5 μm 2.1 × 100 mm. A more detailed method refers to the research of Zhuang et al. (23). Finally, 53 kinds of polyphenols and their derivatives, as well as caffeine and theanine, a total of 55 compounds were identified. These substances are listed in detail in Supplementary Table S1.

### 2.4 Statistical analysis

The sample peak area data are normalized and then used for subsequent analysis (28). The Pearson correlation analysis of polyphenols was performed using the online bioinformatics website^1^ (the *p*-value level was set at 0.05); while the PCoA, hierarchical cluster analysis and Adonis analyses were based on the OmicShare bioinformatics analysis website.^2^ For the content of metal and potentially toxic elements, the one-way analysis of variance (ANOVA) using SPSS (version 22.0) was used to assess the significance of difference (*p* < 0.05). For a single tea sample, the normalized peak area of a specific compound is used as the content index, and for the six major tea categories, the content index is the normalized value of the average peak area of each specific tea compound.

## 3 Results and discussion

### 3.1 Overview of the content of metal and potentially toxic elements in 122 tea samples

Tea is rich in various essential (trace and macro) metal elements for the human body. However, excessive intake of certain metal elements may still cause harm to the human body (10). Here, the contents of ten elements such as Fe, Mg, Al, Cu, Zn, Cr, Pb, As, Ni, and Mn are revealed, and the results are shown in Table 2 and Supplementary Table S2. Among them, the content range of Mg is 1362.17 to 3,848 mg/kg, with an average content of 2135.69 mg/kg; the content range of Al is 170.37 to 2854.66 mg/kg, with an average content of 706.52 mg/kg; the content range of Mn is 259.36 to 2519.20 mg/kg, with an average content of 860.56 mg/kg; the content range of Fe is 51.85 to 595.20 mg/kg, with an average content of 165.62 mg/kg; the content range of Ni is 0.21 to 17.9 mg/kg, with an average range of 6.21 mg/kg; the content range of Cu is 1.34 to 26.56 mg/kg, with an average content of 12.89 mg/kg; the content range of Zn is 6.41 to 74.32 mg/kg, with an average content of 32.63 mg/kg; the content range of Cr is 0.02 to 4.92 mg/kg; the average content is 0.92 mg/kg; the content range of Pb is 0.05 to 2.98 mg/kg, with an average content of 0.79 mg/kg; the content range of As is 0.01 to 0.49 mg/kg, with an average content of 0.23 mg/kg. Zhang et al.’s (13) study in black tea showed that the contents of Fe, Cr, Ni and Mn were 176.60, 4.25, 6.56 and 772.45 mg/kg, respectively, which was similar to our study. However, the contents of Al and Pb were lower than those in Brazilian tea (29).

**Table 2:** The content of 10 metal and potentially toxic elements in different tea categories (mg/kg, LOQ: Limits of quantification).

Tea categories		Mg	Al	Mn	Fe	Ni	Cu	Zn	Cr	Pb	As
		LOQ:3	LOQ:2	LOQ:0.3	LOQ:3	LOQ:0.5	LOQ:0.2	LOQ:2	LOQ:0.2	LOQ:0.05	LOQ:0.005
	Min	1514.34	170.37	366.29	51.85	0.21	4.52	9.36	0.02	0.05	0.01
	Max	3270.29	2110.16	1751.48	418.04	17.9	26.56	60.7	4.91	2.98	0.38
Green tea	Mean	2097.96	520.62	798	156.65	7.64	13.36	35.32	0.88	1.01	0.25
	Median	1973.78	413.9	773.74	142.94	7.37	13.27	35.35	0.34	0.46	0.32
	Number of samples < LOQ	0	0	0	0	0	0	0	10	67	60
	Min	1646.84	264.96	259.36	73.75	0.33	6.43	18.16	0.02	0.22	0.01
	Max	3107.82	1068.65	994.43	182.08	9.5	25.65	74.32	2.26	0.22	0.3
	Mean	2041.41	580.7	633.56	120.21	3.85	14.46	33.9	0.67	0.22	0.13
Black tea	Median	1907.32	499.03	686.16	118.86	3.65	14.18	28.81	0.19	0.22	0.08
	Number of samples < LOQ	0	0	0	0	0	0	0	1	15	11
	Min	1812.78	240.28	604.55	72.81	0.9	1.34	6.41	0.1	0.82	0.08
	Max	3107.55	2327.81	1572.74	237.92	14.63	21.2	59.27	2.93	0.82	0.2
Oolong tea	Mean	2180.13	999.92	952.57	151.46	3.61	9.39	23.55	0.93	0.82	0.14
	Median	2079.53	860.8	969.3	152.55	2.73	9.8	20.79	0.34	0.82	0.13
	Number of samples < LOQ	0	0	0	0	2	0	0	5	14	10
	Min	1935.12	440.06	572.43	115.57	1.9	8.56	21.32	0.19	0.07	0.45
	Max	3848	2854.66	2519.2	595.2	8.7	24.78	33.64	2.55	0.57	0.49
Dark tea	Mean	2658.9	1837.03	1626.42	393.15	5.78	15.79	28.16	1.39	0.38	0.47
	Median	2679.03	2050.43	1677.95	437.48	6.19	15.57	28.26	1.34	0.43	0.47
	Number of samples < LOQ	0	0	0	0	0	0	0	0	4	5
	Min	1362.17	241.81	694.28	76.18	0.77	3.73	20.89	0.5		0.03
	Max	3067.59	1452.77	1002.38	197.75	4.47	14.96	34.72	1.89		0.28
White tea	Mean	2009.62	641.15	799.31	106.86	2.49	9.1	28.26	1.12	N.D.	0.16
	Median	1854.79	465.86	714.05	87.24	2.23	8.85	29.9	0.96		0.16
	Number of samples < LOQ	0	0	0	0	0	0	0	2		3
	Min	1551.69	389.64	449.6	99.7	2.07	9.51	17.35	0.18	0.1	0.15
	Max	2443.28	2623.59	1755.94	339.09	6.27	13.65	40.67	2.65	2.51	0.41
Yellow tea	Mean	2136.24	1086.32	1016.59	175.38	4.54	11.12	29.28	1.07	1.31	0.27
	Median	2193.32	745.48	1016.01	129.13	5.05	10.18	29.85	0.7	1.31	0.25
	Number of samples < LOQ	0	0	0	0	0	0	0	0	4	3

### 3.2 Analysis of the differences in the contents of metals and potentially toxic elements among the six tea categories

**Figure 2:**
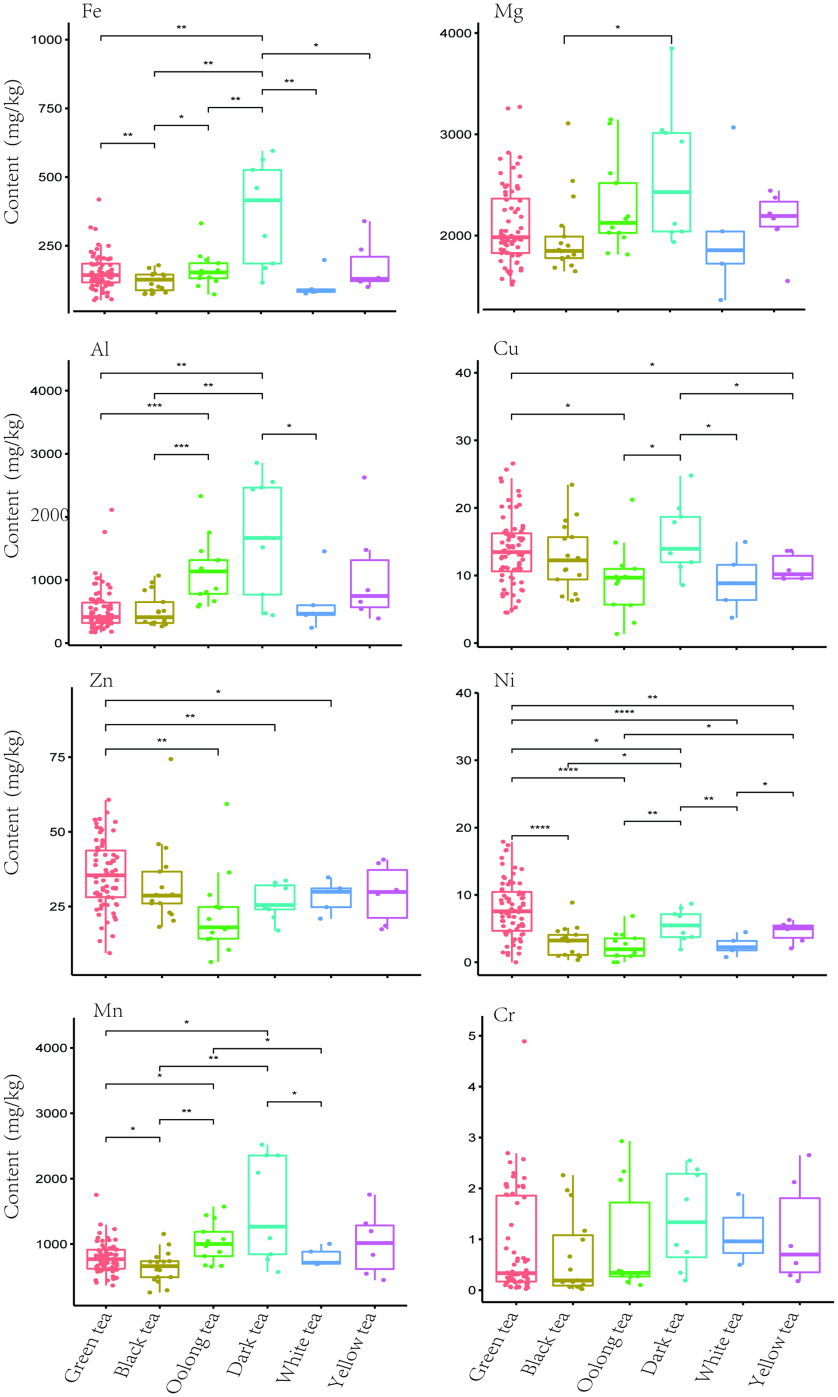
The comparative analysis of metal content among the six tea categories. Significant differences were calculated by one-way ANOVA test (*p < 0.05, **p < 0.01, ***p < 0.001).

In China, traditional tea leaves are divided into six categories according to different processing methods, resulting in huge differences in flavor substances and metal element content (12, 30). As shown in Figure 2, As the trace element with the highest content and demand in the human body, Fe has significant differences among groups such as Green tea vs. Black tea, Green tea vs. Dark tea, and Black tea vs. Oolong tea. Among them, dark tea has the highest content of Fe, which is more suitable for consumers who need to consume more iron; while the content of Zn in green tea is the highest, and it is more suitable for young men to drink. Meanwhile, the contents of elements such as Al, Cu, Zn, Ni, and Mn show rich diversity among different tea types. For example, the above several elements all show significant differences between green tea and oolong tea. The content of Mg only shows a significant difference between black tea and dark tea, while Cr has no obvious difference among different tea types. On the whole, Fe, Mg, Al, Cu, Mn, and Cr all show relatively high levels in dark tea, which may be related to its raw materials and processing techniques. Although the detection rates of Pb and As were low, the detection rate of As in black tea was higher than the overall level. At present, the limit standards for Pb, Cr and As in tea are 5.0 mg/kg, 5.0 mg/kg and 2.0 mg/kg, respectively (NY 659-2003, GB 2762-2022) (31, 32). Therefore, in all samples in this study, the content was lower than the above standard. The latest research shows that the brewing method of green tea may be an important risk factor for aluminum exposure (33). Although Fe, Mg, Al, Zn, Ni, Mn and Cu lack relevant national standards, and moderate tea consumption will not lead to excessive exposure, consumers should still be aware of the potential risks (13, 34).

### 3.3 The variation characteristics of metals and potentially toxic elements content in tea from different provinces

**Figure 3:**
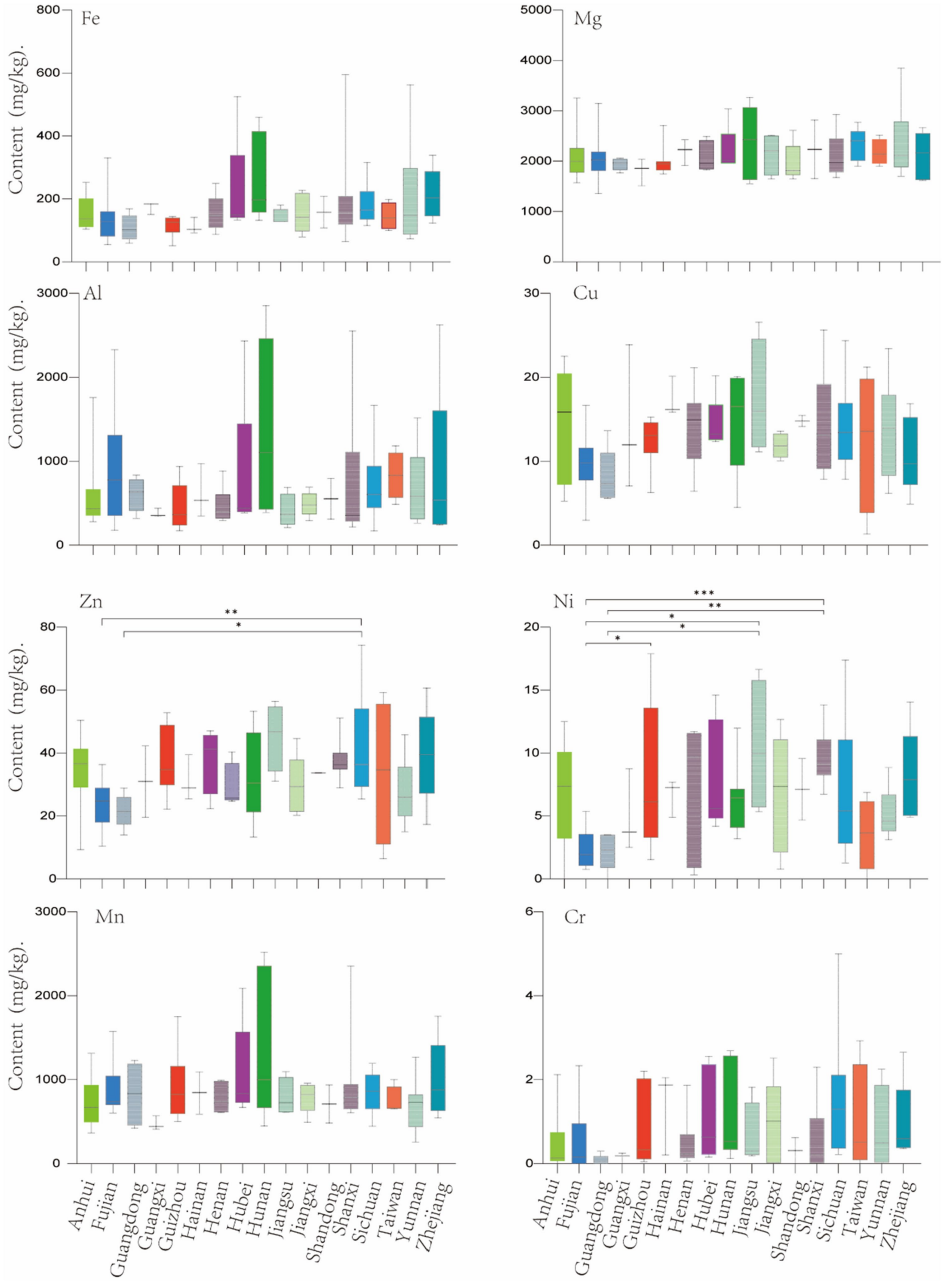
Comparative analysis of metal content in tea from different provinces. Significant differences were calculated by one-way ANOVA test (*p < 0.05, **p < 0.01, ***p < 0.001).

Metal elements in the soil can be absorbed by plants and enter the human body (35). And the characteristics of the soil, field management and the absorption ability of plants can all affect the accumulation level of metal and potentially toxic elements in plants. In this study, we further analyzed the content characteristics of ten elements in tea from different provinces. The results show that there is a significant difference in the Zn content in the tea samples of Fujian and Guangdong provinces when compared with that of Sichuan; the Ni contents of combinations such as Fujian vs. Guizhou, Fujian vs. Jiangsu, Fujian vs. Shanxi, Guangdong vs. Jiangsu and Guangdong vs. Shanxi all showed significant differences. Besides, there is no significant difference in the content of other elements among the various provinces (Figure 3). This may indicate that the processing technique has an important impact on the final content of the metal and potentially toxic elements in the tea. At the same time, it is also implied that under the same drinking style, consumers’ choice of tea products from different provinces has little effect on the intake of elements other than Zn and Ni.

### 3.4 Correlation matrix analysis of compounds

**Figure 4:**
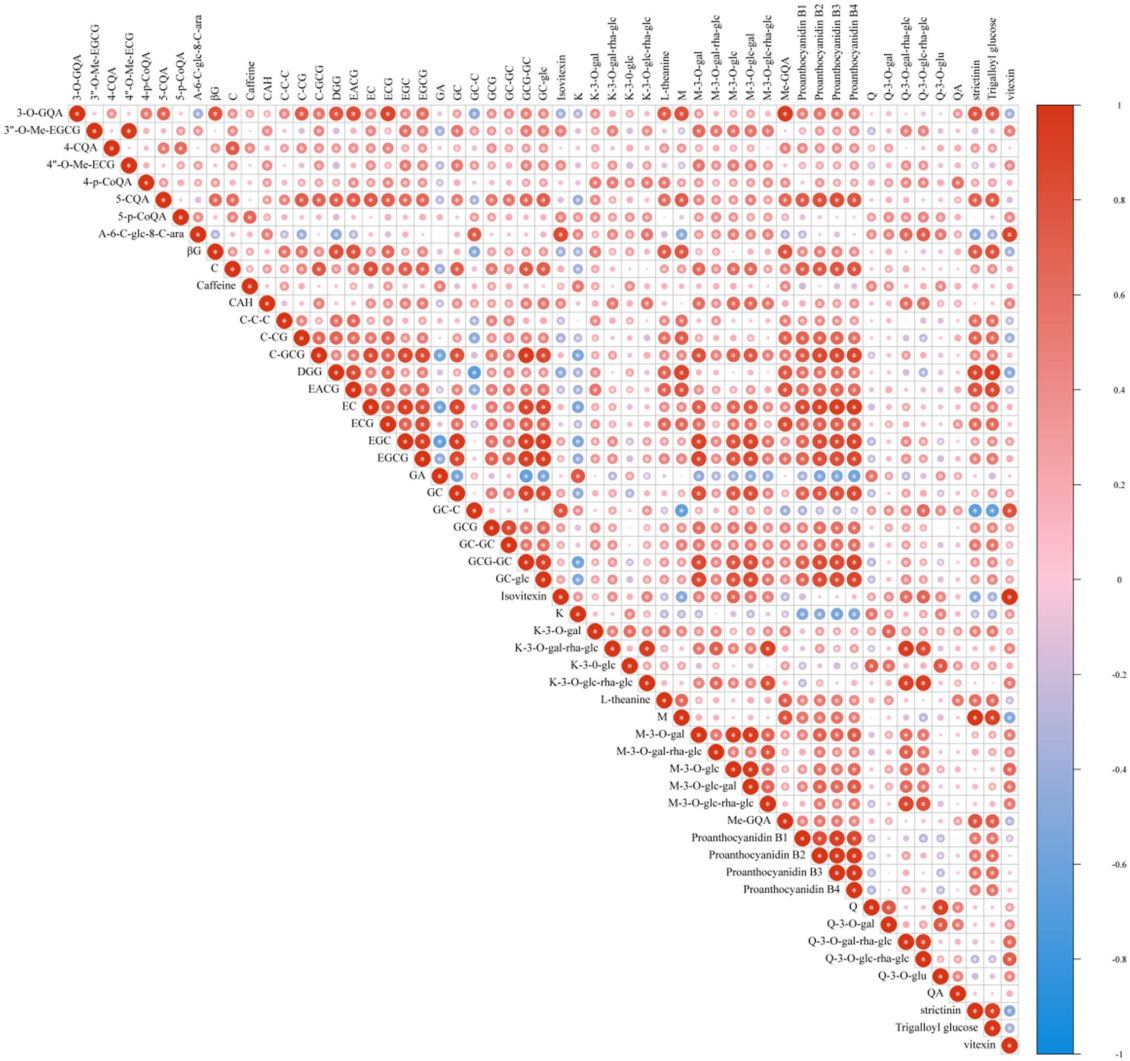
Correlation matrix analysis of polyphenols. The correlation coefficient values range from −1 (colored in blue) to 1 (colored in red).

Tea contains a lot of compounds, which contribute to the bitter taste of tea soup (36, 37). Referring to the method constructed by Zhuang et al. (23), A total of 53 polyphenols were identified in 122 tea samples, as well as two important characteristic compounds, caffeine and theanine. Among these compounds, there are a large amount of substances such as catechins, proanthocyanidins, hydrolysable tannins and flavone glycosides (Detailed substances information was listed in Supplementary Table S1). Further, Pearson correlation analysis was performed to reveal the interrelationships of these substances. The correlation of all secondary generations is shown in Figure 4, with an asterisk in the circle indicating a significant correlation between the two compounds (*p* < 0.05). For example, GA (gallic acid) is considered to be a key precursor of galloacylated catechins, accounting for about 1% of the dry weight of tea, and it is significantly negatively correlated with a variety of catechins and proanthocyanidins (38). Unlike the research results of Zhuang et al. (23), phenolic acid compounds generally show more positive correlations with other substances.

### 3.5 Hierarchical clustering, PCoA and Adonis analysis

**Figure 5:**
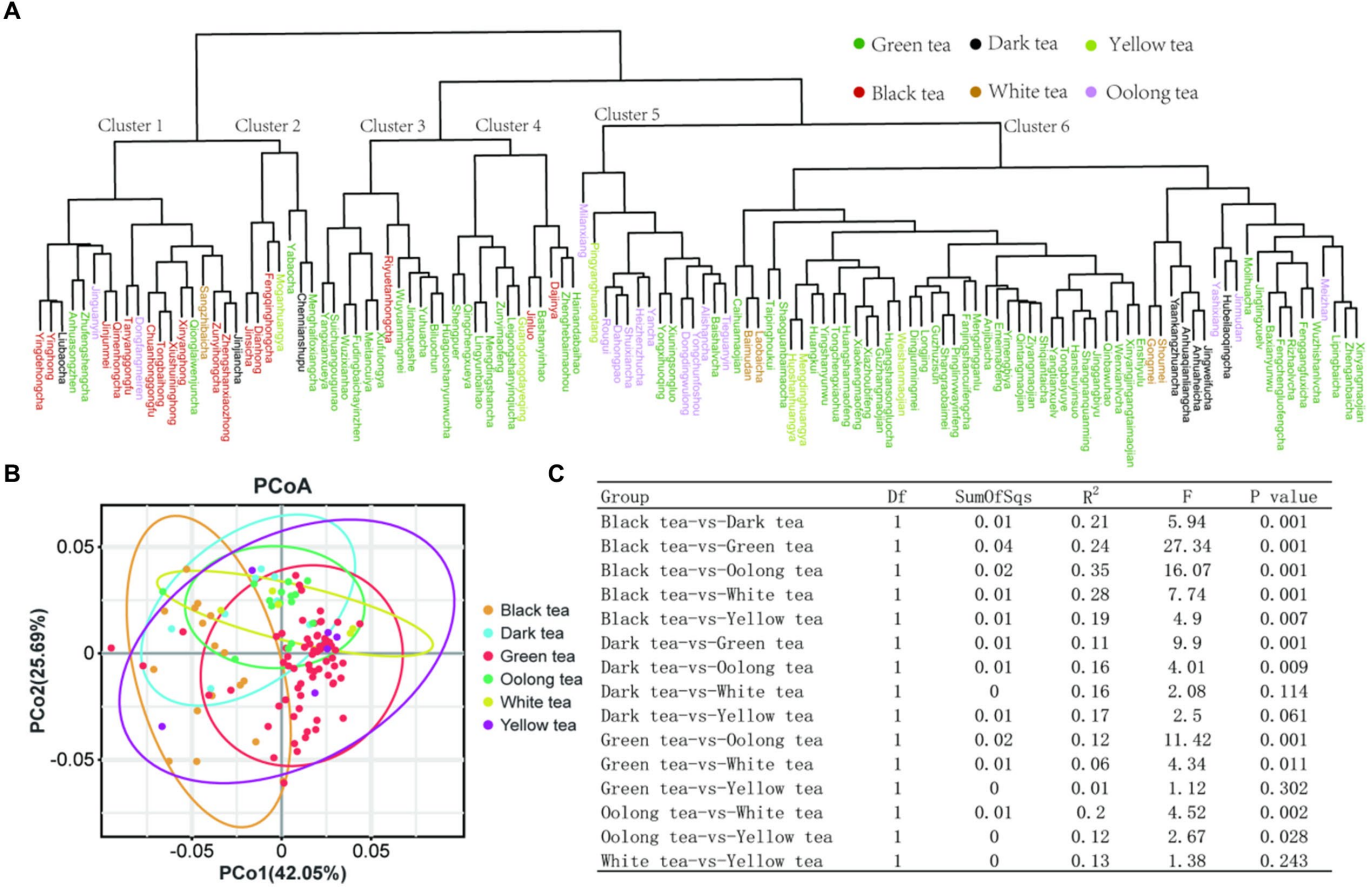
Sample classification analysis based on metabolites. **(A)** Hierarchical cluster analysis; **(B)** PCoA analysis; **(C)** Adonis analysis.

In order to more visually display the influence of the differences of polyphenols and other compounds on tea samples, we carried out hierarchical clustering, PCoA and Adonis analysis (permutation multivariate analysis of variance) based on all compounds. According to the clustering results (Figure 5A), green tea was mainly distributed in clusters 3, 4 and 6, while black tea, oolong tea and dark tea were mainly distributed in clusters 1, 5 and 6, respectively. At the same time, yellow tea and white tea showed no obvious clustering. The reason for such a clustering result may be that different samples used the same tea raw materials or processes. In fact, many white teas and yellow teas may have borrowed from the processing methods of green tea in the processing process. Similar to the result of cluster analysis, in the PCoA analysis, black tea, green tea and oolong tea are clustered more obviously, and the variation explanation rates of the first principal component and the second principal component are 42.05 and 25.69%, respectively, (Figure 5B). In the Adonis analysis, the value of R^2^ shows a positive correlation with the degree of difference among samples. The pairwise analysis of the six tea categories showed that there were no significant differences in polyphenols between the combinations of black tea vs. white tea, dark tea vs. white tea, green tea vs. yellow tea, and white tea vs. yellow tea, which potentially expanded the range of choices of consumers (Figure 5C). In these pairwise comparisons, black tea and oolong tea had the largest difference with an R^2^ of 0.35, while green tea and yellow tea had the smallest difference with an R^2^ of only 0.01. However, it should be noted that this result has certain limitations due to the limitation of the number of samples.

### 3.6 Metabolic spectrum of polyphenols in six tea categories

In order to facilitate the comparison of the relative content of polyphenols in different teas, we normalized the peak area of each metabolite and then calculated the average number in a particular tea category as the content index (CI) based on the number of samples (Supplementary Table S1). Next, we performed a comparative analysis of characteristic polyphenols such as catechin, gallic acid, caffeine, and theanine. The results showed that the contents of caffeine and theanine in white tea were higher, and theanine could reconcile the bitterness of caffeine and improve the freshness of tea (Supplementary Figure S1). In particular, the content of EGCG, C, GCG, EGC, GC and EC in black tea is lower than that of other teas, which may improve the sweet and mellow taste of black tea soup (39). In Table 3, we show in detail the content indices of 55 compounds in different tea categories. For example, black tea has the highest levels of GA (gallic acid), which can lead to an enhanced sour taste of black tea (40). The content of 3”-O-Me-EGCG in oolong tea was higher than that of other teas. Compared with EGCG, the former has higher stability and bioavailability (41). On the whole, the polyphenol content of the six major teas categories from high to low is: white tea, green tea, oolong tea, yellow tea, dark tea and black tea. This result is similar to the study of polyphenol content in tea by Fu et al. (22).

**Table 3:** Compounds content index (CI) of the six major tea categories.

Compounds	Green tea	Black tea	Oolong tea	Dark tea	Yellow tea	White tea
QA	6.52	6.80	6.68	6.36	6.63	6.79
CAH	5.79	5.43	5.89	5.71	5.75	5.93
CQAs						
5-CQA	5.30	4.72	4.82	4.76	4.97	5.24
4-CQA	4.88	4.57	4.78	4.79	4.85	5.22
CoQAs						
5-p-CoQA	5.02	4.99	5.54	5.74	5.44	5.90
4-p-CoQA	5.00	5.04	4.73	4.45	4.88	5.17
GA	6.22	7.02	6.42	7.05	6.73	6.92
GQAs						
3-O-GQA	6.10	6.02	5.67	5.53	5.88	6.09
Me-GQA	4.68	4.59	4.28	4.23	4.53	4.87
Hydrolysable tannins						
βG	6.00	5.79	5.50	5.64	5.74	5.76
Trigallyl glucose	6.40	5.56	5.21	5.61	5.73	5.88
DGG	5.87	5.35	4.83	5.09	5.30	5.38
Strictnin	6.20	5.62	5.00	5.25	5.60	5.93
Galloyllated catechins						
EACG	5.60	5.12	5.16	5.04	5.27	5.42
ECG	7.21	7.04	7.07	6.90	7.11	7.35
GCG	4.40	3.85	4.34	4.26	4.34	4.83
EGCG	7.11	6.28	7.08	6.59	6.85	7.19
Galloyllated PAs						
(E)GCG-(E)GC	5.03	3.70	4.89	4.11	4.51	4.84
(E)C-(E)GCG	4.44	3.54	4.15	3.73	4.03	4.30
(E)C-(E)CG	4.02	3.86	3.59	3.55	3.82	4.08
GC-glc	4.71	3.68	4.73	4.18	4.41	4.74
Catechins						
GC	5.63	4.19	5.76	5.24	5.15	4.95
EGC	6.51	5.07	6.51	5.76	6.01	6.20
C	5.96	5.11	5.97	5.65	5.69	5.78
EC	6.21	5.32	5.97	5.65	5.78	5.77
3″-O-Me-EGCG	3.92	3.10	5.08	3.83	3.92	3.69
4″-O-Me-ECG	4.13	3.56	5.35	4.12	4.19	3.79
PAs						
PA B1	5.18	4.46	4.81	4.52	4.72	4.64
PA B2	4.44	3.66	4.00	3.91	4.05	4.22
PA B3	6.08	5.18	5.64	5.33	5.60	5.76
PA B4	5.39	4.39	4.98	4.79	4.92	5.06
(E)C-(E)C-(E)C	3.53	3.37	3.30	3.47	3.46	3.63
(E)GC-(E)GC	4.46	3.65	4.10	4.32	4.30	4.96
(E)GC-(E)C	4.16	4.38	5.22	5.02	4.86	5.53
K	5.75	6.37	5.93	6.77	6.25	6.45
Q	4.05	4.35	4.04	4.48	4.30	4.58
M	4.83	4.52	3.87	4.15	4.42	4.73
K-Gal(s)						
K-3-0-gal-rha-glc	5.60	4.94	5.30	5.59	5.53	6.24
K-3-0-gal	5.39	5.18	5.24	5.29	5.34	5.58
Q-Gal(s)						
Q-3-O-gal-rha-glc	4.81	4.19	4.74	5.16	4.92	5.70
Q-3-O-gal	5.95	5.96	6.05	6.16	6.11	6.41
M-Gal(s)						
M-3-0-gal-rha-glc	3.65	2.90	3.40	3.61	3.48	3.84
M-3-O-gal	6.00	5.19	6.15	5.89	5.91	6.32
K-Glc(s)						
K-3-0-glc-rha-glc	6.05	5.79	6.09	6.20	6.17	6.73
K-3-0-glc	6.21	6.43	5.89	6.33	6.27	6.48
Q-Glc(s)						
Q-3-0-glc-rha-glc	5.00	4.76	5.40	5.53	5.36	6.12
Q-3-O-glu	6.12	6.37	6.19	6.53	6.39	6.73
M-Glc(s)						
M-3-0-glc-rha-glc	4.36	3.29	4.05	4.24	4.14	4.78
M-3-O-glc-gal	5.07	4.19	5.22	5.03	4.98	5.38
M-3-0-glc	6.11	5.38	6.34	6.15	6.12	6.61
FG(s)						
Isovitexin	4.69	4.31	5.24	5.74	5.11	5.59
A-6-C-glc-8-C-ara	5.54	5.45	5.73	6.26	5.82	6.14
Vitexin	4.83	4.48	5.41	5.88	5.28	5.78
Others						
Caffeine	7.65	7.83	7.76	8.04	7.88	8.10
L-theanine	5.43	5.36	4.99	4.93	5.27	5.62

More detailed compound information can be viewed in Supplementary Table S1.

## 4 Conclusion

In this study, a total of 122 tea samples from 20 provinces were collected, and the contents of ten elements such as Fe, Mg, Zn, Al, Cu, Ni, Mn, Cr, Pb, and As were detected. The results showed that the changes in metal content between different tea categories were more significant than those in different production regions. And the metal content in all tea samples conforms to the national limit standards. Ordinary consumers’ moderate consumption of tea will not cause potential over-exposure risks. Dark tea and green tea are more suitable for consumers who need to consume more Fe and Zn, respectively. Meanwhile, in addition to caffeine and theanine, we mainly detected 53 kinds of polyphenols in these samples. The analysis found that there were different degrees of differences between these substances in different tea categories. Among them, green tea and yellow tea have the least difference, while dark tea and oolong tea have the greatest difference. Therefore, in some cases, consumers can try to replace green tea with yellow tea for drinking. The polyphenol content from high to low is white tea, green tea, oolong tea, yellow tea, dark tea and black tea. The lower content of phenolic substances is suitable for consumers who prefer a milder bitter and astringent taste. In conclusion, this study provides a more comprehensive perspective to understand the quality characteristics of commercially available tea, and provides data support for the rational choice of consumers. And also lay a foundation for conducting risk assessment in the future based on the contents of these elements/compounds and more extensive consumer data.

## Data Availability

The original contributions presented in the study are included in the article/Supplementary material, further inquiries can be directed to the corresponding authors.

## Footnotes

^1^ https://www.bioinformatics.com.cn/

^2^ https://www.omicshare.com/tools/
