# Supplementary figures and images for: Extensive ICP-MS and HPLC-QQQ detections reveal the content characteristics of main metallic elements and polyphenols in the representative commercial tea on the market
Source: Front Nutr. 2024 Aug 12;11:1450348. doi: 10.3389/fnut.2024.1450348 (PMC11345263; doi:10.3389/fnut.2024.1450348)

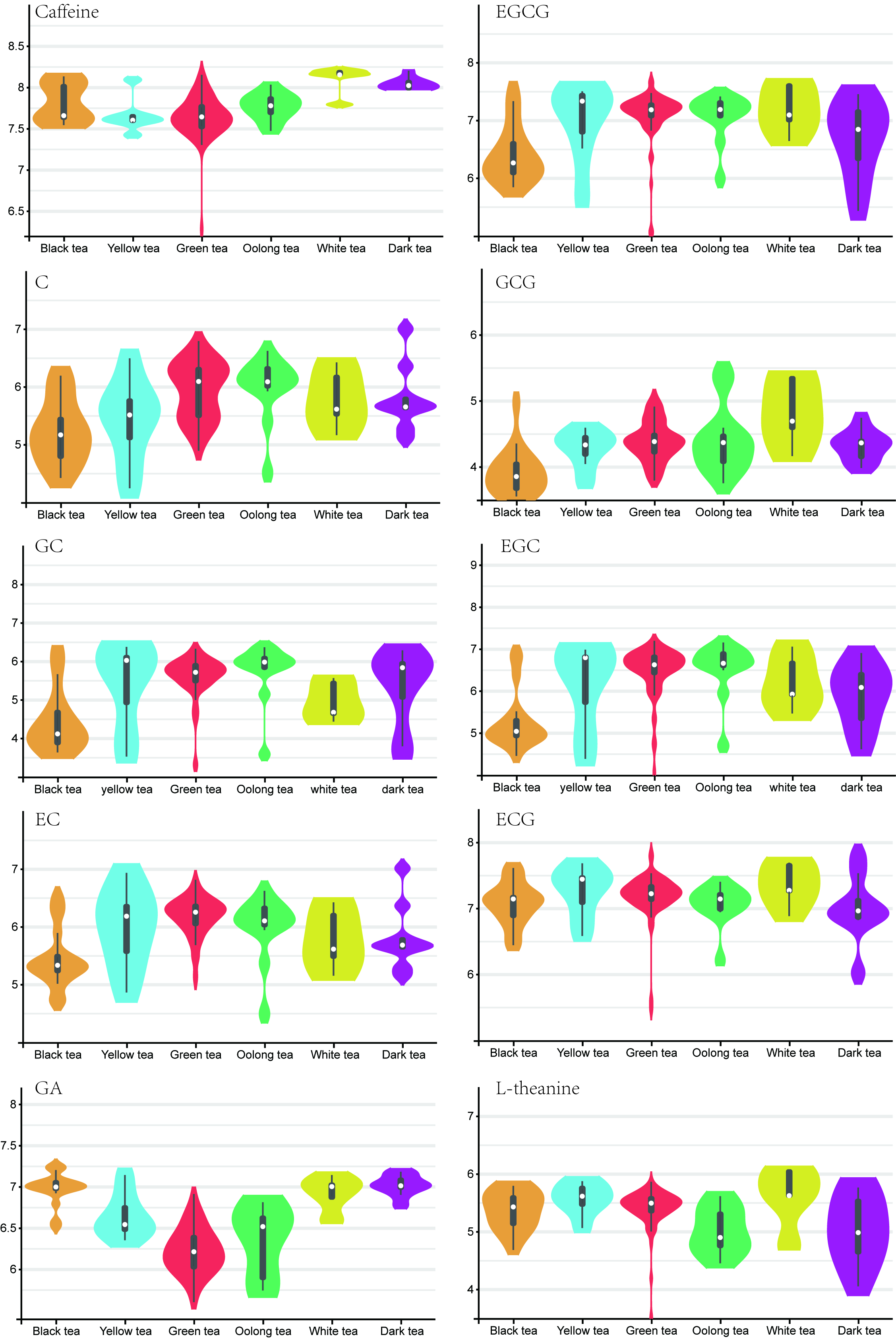

Supplement: Supplementary file 1 [file Image_1.tif]
