# Supplementary material for: Extensive ICP-MS and HPLC-QQQ detections reveal the content characteristics of main metallic elements and polyphenols in the representative commercial tea on the market
Source: Front Nutr. 2024 Aug 12;11:1450348. doi: 10.3389/fnut.2024.1450348 (PMC11345263; doi:10.3389/fnut.2024.1450348)
Supplement: Supplementary file 3 [file Table_2.doc]

Table S2 the content of 10 metal and potentially toxic elements in 122 tea samples (mg/kg)

| **Name** | **Mg** | **Al** | **Mn** | **Fe** | **Ni** | **Cu** | **Zn** | **Cr** | **Pb** | **As** |
| --- | --- | --- | --- | --- | --- | --- | --- | --- | --- | --- |
| Alishancha | 2517.83±157.45 | 808.79±60.56 | 654.66±43.75 | 198.31±11.52 | 4.1±1.13 | 11.44±0.86 | 24.83±4.52 | 0.38±0.07 | N.D. | 0.13±0.01 |
| Anhuaheicha | 2429.63±66.79 | 2464.69±97.73 | 2355.43±64.56 | 415.31±13.22 | 6.87±0.05 | 19.92±0.27 | 21.32±0.32 | 2.26±0.03 | 0.07±0.08 | N.D. |
| Anhuaqianliangcha | 3013.01±219.58 | 2854.66±204.94 | 2519.2±175.92 | 459.65±33.54 | 7.17±1.1 | 17.88±1.32 | 24.04±2.96 | 0.34±0.06 | 0.57±0.81 | 0.47±0.02 |
| Anhuasongzhen | 3270.29±331.7 | 878.49±75.21 | 833.28±81.39 | 179.99±12.92 | 6.47±0.58 | 16.51±0.55 | 53.3±11.04 | 2.69±0.13 | N.D. | 0.09±0.02 |
| Anjibaicha | 1645.6±68.34 | 255.67±9.49 | 880.36±16.55 | 123.74±1.41 | 8.6±0.71 | 4.91±0.75 | 37.17±2.49 | 0.36±0.07 | N.D. | N.D. |
| Baimudan | 1722.64±25.95 | 465.86±249.45 | 714.05±13.09 | 81.73±2.67 | 3.2±0.08 | 8.85±0.16 | 29.9±0.53 | 0.96±0.19 | N.D. | N.D. |
| Baishalvcha | 2429.09±4.39 | 535.77±19.4 | 587.97±5.46 | 141.71±4.64 | 7.27±0.09 | 16.16±0.48 | 28.94±1.06 | 1.87±0.05 | N.D. | N.D. |
| Bashanyinhao | 1546.5±56.86 | 223.37±7.03 | 617.17±20.6 | 77.94±6.35 | 3.4±0.49 | 10.86±1.26 | 28.15±3.75 | N.D. | N.D. | N.D. |
| Baxianyunwu | 1965.59±97.52 | 2110.16±2204.57 | 759.1±38.83 | 162.98±9.18 | 8.5±0.16 | 17.34±0.26 | 35.46±1.65 | 2.3±0.24 | N.D. | N.D. |
| Biluochun | 1675.95±69.39 | 1106.82±50.31 | 1298.3±42.46 | 310.78±7.87 | 4.1±0.22 | 4.52±0.26 | 13.38±0.74 | 0.13±0.03 | N.D. | N.D. |
| Caihuamaojian | 1958.35±15.26 | 386.88±5.96 | 836±2.17 | 133.86±4.5 | 5.63±0.05 | 13.23±0.11 | 25.76±0.14 | 0.16±0.02 | N.D. | N.D. |
| Chennianshupu | 3848±32.74 | 1515.41±27.44 | 1266.17±19.82 | 563±11.06 | 8.03±0.19 | 18.67±0.25 | 33.02±0.44 | 1.78±0.02 | N.D. | 0.45±0.01 |
| Chuanhonggongfu | 2539.35±86.44 | 650.7±44.43 | 852.84±17.92 | 145.08±13.49 | 5.1±1.59 | 19.03±0.2 | 74.32±25.07 | 0.4±0.1 | N.D. | 0.07±0.05 |
| Dahongpao | 2026.25±211.54 | 1750.46±175.25 | 1398.81±141.39 | 134.92±14.62 | 2.73±0.25 | 9.81±0.36 | 24.55±1.09 | 0.27±0.02 | N.D. | 0.18±0.01 |
| Dajinya | 1703.93±20.07 | 264.96±4.35 | 293.17±2.93 | 95.93±1.08 | 4.07±0.09 | 7.24±1.07 | 22.93±0.73 | N.D. | N.D. | N.D. |
| Dianhong | 1924.15±15.86 | 304.73±14.54 | 801.63±12.69 | 110.23±3.47 | 4.93±0.25 | 17.17±0.27 | 45.86±5.91 | 2.26±0.03 | N.D. | 0.08±0.01 |
| Dingjunmingmei | 1803.4±19.91 | 219.33±5.72 | 842.71±9.67 | 148.86±3.77 | 6.73±0.05 | 8.78±0.17 | 39.53±0.58 | 0.17±0.02 | N.D. | N.D. |
| Dongdingwulong | 2125.39±54.67 | 1183.04±18.2 | 1001.19±15.61 | 158.38±3.37 | N.D. | 1.34±0.13 | 6.41±0.64 | N.D. | N.D. | N.D. |
| Dongfangmeiren | 2165.16±124.28 | 860.8±71.35 | 666.05±42.92 | 121±12.18 | 6.9±1.13 | 21.2±0.39 | 59.27±23.17 | 2.93±0.54 | N.D. | 0.08±0.04 |
| Emeimaofeng | 2758.06±80.66 | 559.48±21.31 | 445.87±15.05 | 316.52±7.63 | 9.23±0.09 | 24.37±0.71 | 54.04±0.48 | 0.57±0.01 | N.D. | N.D. |
| Enshiyulu | 1958.56±19.81 | 441.47±3.99 | 671.86±7.57 | 149.43±4.4 | 4.17±0.05 | 12.34±0.07 | 25.5±0.5 | 0.63±0.03 | N.D. | N.D. |
| Fanjingshancuifengcha | 1988.36±6.4 | 241.8±6.03 | 1045.53±8.78 | 138.68±4.48 | 11.67±0.21 | 14.87±0.09 | 52.84±0.98 | 1.28±0.07 | N.D. | N.D. |
| Fengchengluofengcha | 1814.77±36.03 | 540.78±31.84 | 913.22±21.67 | 141.85±3.21 | 9.5±0.33 | 12.99±0.31 | 31.2±2.71 | 0.05±0.02 | N.D. | N.D. |
| Fenggangfuxicha | 1929.06±58.05 | 488.67±15.09 | 609.69±13.5 | 123.71±2.48 | 6.13±0.42 | 10.79±1.06 | 34.79±2.35 | 2.02±0.14 | N.D. | N.D. |
| Fengqinghongcha | 3107.82±217.44 | 1068.65±86.29 | 730.63±39.17 | 178.55±10.63 | 8.87±0.19 | 23.41±0.47 | 38.26±0.72 | 0.07±0.06 | 0.22±0.31 | 0.21±0.01 |
| Fudingbaichayinzhen | 2073.85±21.41 | 179.79±6.91 | 700.63±9.8 | 54.76±0.97 | 5.37±0.31 | 7.77±0.52 | 35.41±2.35 | N.D. | N.D. | N.D. |
| Gongmei | 1854.79±59.58 | 445.87±12.64 | 884.97±19.6 | 76.18±4.27 | 0.77±0.09 | 6.37±0.54 | 20.89±1.13 | N.D. | N.D. | N.D. |
| Guangdongdayeqing | 2061.84±10.52 | 837.29±97.87 | 834.97±9.7 | 99.7±2.03 | 2.07±0.21 | 13.65±0.35 | 18.51±0.33 | 0.18±0.03 | N.D. | N.D. |
| Guzhangmaojian | 1633.72±92.55 | 428.35±20 | 665.22±29.1 | 157.56±8.52 | 12±1.49 | 20.12±2.86 | 46.52±5.9 | 2.57±0.35 | N.D. | N.D. |
| Guzhuzisun | 2670.58±129.1 | 582.16±16.66 | 1060.23±58.37 | 204.56±11.4 | 14.03±0.4 | 16.86±0.46 | 60.7±10.01 | 0.39±0.01 | N.D. | 0.18±0.01 |
| Hainandabaihao | 2230.8±80.63 | 348.99±8.33 | 848.41±28 | 103.36±2.14 | 4.9±0.14 | 20.15±0.45 | 39.48±0.98 | 2.05±0.07 | N.D. | 0.34±0.01 |
| Hanshuiyinsuo | 1942.43±11.92 | 291.4±3.21 | 906.34±6.05 | 120.64±1.04 | 9.87±0.38 | 7.87±0.37 | 28.99±1.27 | N.D. | N.D. | N.D. |
| Heizhenzhucha | 2353.4±59.08 | 1025.74±182.09 | 604.55±1.42 | 237.92±23.93 | 1.4±0.01 | 11.07±0.09 | 17.67±0.34 | 0.37±0.01 | N.D. | N.D. |
| Huaguoshanyunwucha | 1648.3±33.36 | 377.39±6.08 | 626.4±9.81 | 127.43±2.84 | 6.8±0.22 | 13.31±0.56 | 31.13±1.56 | 0.19±0.05 | N.D. | N.D. |
| Huangkui | 2185.3±106.22 | 435.38±18.81 | 916.41±35.11 | 111.53±3.64 | 10.17±0.41 | 15.38±0.59 | 41.37±1.76 | 0.14±0.1 | N.D. | N.D. |
| Huangshanmaofeng | 1998.73±37.81 | 665.04±397.02 | 767.32±18.47 | 221.94±57.49 | 12.53±0.09 | 16.35±0.13 | 43.96±0.08 | 0.74±0.08 | 0.46±0.03 | N.D. |
| Huangshansongluocha | 1964.81±83.22 | 417.52±18.57 | 670.84±26.39 | 136.75±6.01 | 6.13±0.34 | 6.9±4.02 | 29.64±2.18 | N.D. | N.D. | N.D. |
| Hubeilaoqingcha | 3041.51±206.01 | 2434.06±150.73 | 2089.73±119.46 | 525.8±27.92 | 5.5±0.08 | 13.26±0.14 | 24.69±0.72 | 2.55±0.01 | N.D. | N.D. |
| Huoshanhuangya | 2217.74±305.4 | 653.66±101.13 | 1315.7±193.05 | 119.35±20.1 | 5.57±0.19 | 9.59±0.34 | 29.17±0.72 | 2.12±0.09 | N.D. | 0.15±0.02 |
| Jinggangbiyu | 2584.75±41.42 | 392.29±2.45 | 1066.79±5.5 | 202.69±1.29 | 13.8±0.29 | 15.16±0.34 | 37.14±2.3 | 2.08±0.06 | N.D. | 0.32±0.02 |
| Jingtingxuelv | 2262.03±174.12 | 356.41±90.98 | 466.62±34.14 | 109.06±7.09 | 8.07±0.09 | 21.8±0.04 | 50.44±0.86 | 0.07±0.02 | N.D. | N.D. |
| Jinguanyin | 3107.55±34.98 | 779.43±6.9 | 674.24±5.03 | 211.1±5.97 | 4.17±0.17 | 14.86±0.13 | 28.85±0.96 | 2.33±0.02 | N.D. | N.D. |
| Jingweifucha | 2928.43±159.97 | 2552.22±152 | 2353.94±135.67 | 595.2±32.85 | 8.7±0.08 | 24.78±0.37 | 33.64±0.66 | 0.75±0.08 | 0.33±0.02 | 0.49±0.01 |
| Jinjiancha | 2035.44±133.66 | 768.38±121.96 | 764.72±44.72 | 115.57±7.17 | 1.9±0.14 | 11.27±0.31 | 32.08±1.91 | 2.37±0.1 | N.D. | N.D. |
| Jinjunmei | 1812±48.06 | 354.46±5.04 | 738.85±2.49 | 127.48±1.16 | 1.07±0.17 | 12.58±0.25 | 29±0.4 | 0.11±0.06 | N.D. | N.D. |
| Jinluo | 1990.59±61.97 | 323.82±11.04 | 602.19±19.82 | 148.45±2.76 | 3.13±0.05 | 12.92±2.16 | 31.38±1.57 | 1.96±0.04 | N.D. | N.D. |
| Jinmudan | 2189.82±74.77 | 1316.53±39.15 | 1572.74±67.71 | 186.45±24.79 | 3.57±0.56 | 10.99±0.36 | 36.38±9.7 | 0.16±0.02 | N.D. | 0.11±0.03 |
| Jinsicha | 1848.78±21.19 | 836.36±850.96 | 259.36±8.8 | 73.75±5.15 | 4.63±0.21 | 9.4±0.45 | 26.02±1.51 | 0.06±0.06 | N.D. | N.D. |
| Jintanqueshe | 2479.43±62.3 | 212.13±8.14 | 823.8±24.36 | 129.11±4.74 | 16.67±0.12 | 11.15±0.07 | 49.83±0.98 | 1.82±0.01 | N.D. | 0.32±0.02 |
| Laobaicha | 1362.17±40.17 | 241.81±13.13 | 694.28±27.98 | 91.42±3.96 | 1.77±0.45 | 3.73±1 | 24.75±3.89 | N.D. | N.D. | N.D. |
| Leigongshanyinqiucha | 2709.71±271.06 | 927.8±73.78 | 504.49±51.43 | 144.02±13.5 | 2.13±0.26 | 13.07±0.38 | 31.13±1.28 | 2.2±0.06 | N.D. | N.D. |
| Lingyunbaihao | 1514.34±24.75 | 345.64±24.21 | 443.46±4.9 | 150.92±3.32 | 2.53±0.29 | 7.05±0.95 | 19.6±3.26 | N.D. | N.D. | N.D. |
| Lipingbaicha | 1951.12±10.84 | 247.28±3.81 | 827.09±3.57 | 130.66±2.22 | 9.37±0.05 | 15.25±0.02 | 46.29±0.58 | 0.15±0.02 | N.D. | N.D. |
| Liubaocha | 2040.08±36.8 | 440.06±8.64 | 572.43±6.7 | 185.18±3.45 | 3.77±0.24 | 11.96±0.33 | 31.05±0.57 | 0.19±0.03 | N.D. | N.D. |
| Longjing | 1619.9±15.2 | 243.49±8.58 | 716.91±19.66 | 167.53±2.22 | 7.9±0.24 | 9.7±0.57 | 42.24±1.5 | 0.6±0.03 | N.D. | N.D. |
| Meitancuiya | 1997.9±100.4 | 237.31±11.3 | 575.37±24.68 | 92.6±4.77 | 17.9±0.49 | 11.22±0.33 | 51.53±1.73 | 2.04±0.18 | N.D. | 0.01±0.01 |
| Meizhan | 1827.71±43.73 | 240.28±15.82 | 693.83±6.78 | 105.66±2.41 | 1.07±0.09 | 4.48±0.46 | 25.31±0.8 | N.D. | N.D. | N.D. |
| Mengdingganlu | 1903.22±58.29 | 333.1±8.15 | 1049.28±7.63 | 201.45±10.47 | 17.4±0.29 | 16.2±0.34 | 50.59±0.94 | 0.22±0.03 | N.D. | N.D. |
| Mengdinghuangya | 2374.01±161.21 | 1474.66±403.07 | 1197.04±86.04 | 125.69±9.06 | 6.27±0.26 | 10.77±0.29 | 40.67±3.18 | 0.29±0.04 | N.D. | 0.25±0.01 |
| Mengdingshancha | 2773.89±69.14 | 560.39±8.34 | 475.58±6.62 | 203.87±1.42 | 5.7±0.92 | 7.86±0.11 | 30.94±0.15 | 2.03±0.01 | N.D. | N.D. |
| Menghaifoxiangcha | 2139.85±186.3 | 1027.65±78.94 | 774.34±64.24 | 418.04±32.62 | 4.63±0.37 | 15.71±1.24 | 25.65±2.14 | 0.82±0.08 | N.D. | N.D. |
| Milanxiang | 1825.04±55.69 | 578.81±30.95 | 1187.65±22.21 | 103.05±9.96 | N.D. | 5.69±3.78 | 14.01±3.14 | N.D. | 0.82±1.16 | N.D. |
| Moganhuangya | 2443.28±135.47 | 539.05±30.05 | 546.28±29.95 | 235.9±12.67 | 5.17±0.05 | 9.51±6.04 | 39.47±0.8 | 2.65±0.07 | N.D. | 0.41±0.02 |
| Molihuacha | 1968.31±41.71 | 783.93±169.31 | 1228.49±11.85 | 135.58±15.8 | 3.07±0.25 | 11.01±0.49 | 21.45±7.43 | 0.3±0.14 | 2.98±3.81 | N.D. |
| Pinglinvwayinfeng | 2268.27±27.06 | 308.76±6.94 | 822.74±1.81 | 118.03±0.33 | 11.77±0.39 | 9.55±0.38 | 36.19±1.27 | 0.22±0.01 | N.D. | N.D. |
| Pingyanghuangtang | 2168.9±47.54 | 2623.59±14.34 | 1755.94±7.51 | 339.09±5.54 | 4.93±0.12 | 13.63±0.22 | 17.35±0.32 | 0.87±0.16 | 0.1±0.03 | N.D. |
| Qimenhongcha | 1777.37±59.05 | 306.09±13.55 | 496.3±23.74 | 143.66±7.71 | 3.23±0.54 | 18.12±3.17 | 36.66±5.83 | 0.16±0.07 | N.D. | N.D. |
| Qinbawuhao | 1675.24±27.07 | 321.84±6.66 | 607.32±12 | 137.87±6.79 | 8.73±0.66 | 16.99±1.52 | 41.54±3.38 | 0.02±0.03 | N.D. | N.D. |
| Qingchengxueya | 2434.43±109.13 | 660.68±27.41 | 713.63±67.53 | 180.08±30.9 | 3.17±0.05 | 14.13±0.17 | 29.88±0.31 | 1.89±0.03 | N.D. | N.D. |
| Qintangmaojian | 1862.28±132.53 | 354.62±27.81 | 410.93±31.67 | 184.53±13.72 | 8.77±0.31 | 23.9±0.66 | 42.33±0.94 | 0.25±0.03 | N.D. | N.D. |
| Qionglaiwenjuncha | 2488.71±149.85 | 483.13±32.4 | 947.28±59.06 | 149.87±7.38 | 1.27±0.17 | 14.48±1.77 | 27.91±1.46 | 1.71±0.06 | N.D. | N.D. |
| Riyuetanhongcha | 1900.11±29.08 | 488.34±88.48 | 663.25±6.06 | 100.51±1.31 | 3.23±0.12 | 15.67±0.2 | 44.61±0.53 | 0.66±0.11 | N.D. | N.D. |
| Rizhaolvcha | 2818.34±115.23 | 795.38±24.39 | 938.2±28.05 | 208.77±5.84 | 4.7±0.22 | 14.13±0.27 | 33.68±2.34 | 0.62±0.09 | N.D. | 0.21±0.01 |
| Rougui | 2028.54±36.5 | 607.89±22.9 | 815.39±12.24 | 131.55±2.57 | 3.13±0.17 | 9.68±0.35 | 20.79±0.53 | 0.31±0.02 | N.D. | N.D. |
| Sangzhibaicha | 3067.59±136.89 | 1452.77±852.9 | 1002.38±50.38 | 197.75±7.86 | 4.47±0.09 | 14.96±0.21 | 31.04±0.66 | 0.5±0.24 | N.D. | 0.28±0.02 |
| Shangnanquanming | 1992.49±61.74 | 388.33±1.73 | 651.87±8.52 | 227.16±2.86 | 10.87±0.31 | 9.25±0.45 | 35.29±1.4 | 0.56±0.03 | N.D. | N.D. |
| Shangraobaimei | 1979.24±9.42 | 480.16±49.32 | 775.38±9.45 | 210.71±2.34 | 12.67±0.21 | 13.59±0.19 | 44.63±1.53 | 1.02±0.06 | N.D. | N.D. |
| Shaoguanbaimaocha | 2045.3±29.96 | 637.01±216.33 | 823.78±3.77 | 59.59±1.85 | 2.3±0.08 | 7.32±0.24 | 24.25±0.75 | 0.06±0.04 | N.D. | N.D. |
| Shengpuer | 2115.93±83.3 | 471.75±14.12 | 843.86±13.52 | 168.53±4.32 | 3.53±0.09 | 13.94±8.3 | 16.94±0.31 | 0.5±0.04 | N.D. | N.D. |
| Shiqiantaicha | 1998.06±24.9 | 365.92±5.14 | 762.9±7.12 | 141.57±1.7 | 5.8±0.01 | 14.34±0.23 | 31.73±0.28 | 0.33±0.02 | N.D. | N.D. |
| Shoumei | 2040.9±29.7 | 599.42±20.85 | 700.86±15.63 | 87.24±1.9 | 2.23±0.05 | 11.58±0.23 | 34.72±0.5 | 1.89±0.06 | N.D. | 0.03±0.02 |
| Shuixiancha | 2079.53±131.32 | 1135.84±61.11 | 1045.43±67.45 | 152.63±9.37 | 1.93±0.26 | 9.15±0.24 | 16.23±0.66 | 0.27±0.07 | N.D. | N.D. |
| Suichuangougunao | 1803.4±19.09 | 447.56±3.09 | 959.83±5.46 | 116.75±0.28 | 3.43±0.17 | 11.84±0.7 | 22.65±1.04 | N.D. | N.D. | N.D. |
| Taipinghoukui | 1571.16±39.22 | 282.36±3.68 | 660.34±7.36 | 104.56±1.03 | 1.43±0.09 | 7.25±0.25 | 23.9±0.53 | 0.1±0.01 | N.D. | N.D. |
| Tanyanggongfu | 2096.85±13.84 | 961.58±452.17 | 723.9±7.67 | 74.09±3.56 | 3.73±0.05 | 10.79±0.28 | 18.16±0.81 | 0.04±0.06 | N.D. | N.D. |
| Tieguanyin | 2614.77±103.19 | 2327.81±79.59 | 969.3±40.12 | 144.99±6.67 | 0.97±0.17 | 9.8±0.26 | 18.01±2.85 | 0.1±0.04 | N.D. | 0.2±0.03 |
| Tongbaihong | 1846.42±79.64 | 887.24±672.98 | 610.18±34.21 | 87.93±4.46 | 1.1±0.01 | 15.44±0.06 | 28.61±0.86 | 1.87±0.15 | N.D. | N.D. |
| Tongbaiyuye | 2495.36±206.12 | 392.22±28.2 | 980.5±66.54 | 185.6±12.74 | 11.57±0.17 | 14.41±0.26 | 45.23±0.42 | 0.27±0.02 | N.D. | 0.33±0.02 |
| Tongchengxiaohua | 1734.91±121.24 | 399.82±66.94 | 366.29±22.86 | 200.79±19.25 | 10.07±0.57 | 22.5±0.38 | 40.26±0.64 | 0.3±0.06 | N.D. | N.D. |
| Weishanmaojian | 1551.69±175.24 | 389.64±39.96 | 449.6±30.8 | 132.56±12.03 | 3.23±0.33 | 9.55±1.05 | 30.52±9.44 | 0.53±0.07 | 2.51±3.3 | N.D. |
| Wenxianlvcha | 1825.9±53.8 | 370.98±4.96 | 770.79±15.44 | 144.63±4.84 | 6.73±0.56 | 10.03±0.26 | 47.25±30.49 | 1.88±0.04 | 1.2±1.7 | N.D. |
| Wuyuanmingmei | 2616.3±131.87 | 694.07±26.14 | 823.38±34.17 | 227.39±8.68 | 7.4±0.08 | 10.88±0.57 | 29.38±1.18 | 2.51±0.03 | N.D. | N.D. |
| Wuzhishanlvcha | 1916.79±22.12 | 972.3±217.58 | 1093.65±5.38 | 92.49±1.39 | 7.7±0.92 | 15.83±0.73 | 25.48±0.15 | 0.21±0.02 | N.D. | N.D. |
| Wuzixianhao | 1728.58±44.48 | 263.02±4.88 | 646.58±7.92 | 65.17±23.24 | 7.47±0.25 | 11.06±0.67 | 36.54±2.51 | N.D. | N.D. | N.D. |
| Xiaokengmaofeng | 2463.19±104.56 | 545.14±26.08 | 947.99±40.73 | 200.71±9.41 | 8.13±0.12 | 20.45±0.5 | 40.19±0.71 | 2.08±0.1 | N.D. | N.D. |
| Xiazhoubifeng | 2017.83±79.46 | 459.13±6.42 | 787.16±17.89 | 148.16±2.72 | 10.73±0.69 | 20.19±1.09 | 40.39±2.66 | 0.27±0.12 | N.D. | N.D. |
| Xinyanghong | 2385.97±52.05 | 509.71±7.21 | 994.43±10.83 | 142.05±2.2 | 0.33±0.05 | 6.43±0.88 | 22.36±0.83 | 0.16±0.01 | N.D. | N.D. |
| Xinyangjingangtaimaojian | 1827.95±91.87 | 329.18±16.8 | 613.56±26.41 | 116.13±5.09 | 8.97±1.19 | 21.14±3.01 | 47.12±5.43 | 0.31±0.1 | N.D. | N.D. |
| Xinyangmaojian | 1884.37±44.53 | 296.72±3.56 | 710.3±5.04 | 154.3±0.96 | 10.4±1.91 | 11.6±0.32 | 40.34±0.32 | 0.06±0.01 | N.D. | N.D. |
| Xiuningsongluo | 3255.25±56.48 | 691.4±15.87 | 665.47±15.22 | 253.93±4.86 | 7.33±0.12 | 15.86±0.26 | 30.53±1.08 | 0.07±0.04 | N.D. | N.D. |
| Xiushuininghong | 1646.84±122.91 | 293.97±6.28 | 494.29±30.35 | 79.08±4.56 | 0.83±0.05 | 10.05±0.16 | 20.21±0.38 | 1.17±0.11 | N.D. | 0.01±0.01 |
| Xufulongya | 2253.55±39.22 | 170.37±1.44 | 873.65±10 | 138.67±3.74 | 16.6±0.08 | 12.76±0.2 | 54.29±0.57 | 4.94±0.04 | N.D. | 0.36±0.02 |
| Yaankangzhuancha | 1935.12±26.03 | 1666.79±81.91 | 1089.75±11.53 | 285.45±4.87 | 4.33±0.12 | 8.56±0.07 | 25.46±0.45 | 0.89±0.09 | 0.53±0.07 | N.D. |
| Yabaocha | 2472.24±52.57 | 580.72±11.01 | 582.43±6.2 | 79.07±1.46 | 5.37±0.05 | 6.2±0.25 | 15.04±0.45 | N.D. | N.D. | N.D. |
| Yancha | 1812.78±72.21 | 1310.48±6.39 | 1075.97±3.91 | 160.65±2.28 | 1.37±0.05 | 2.99±0.17 | 10.46±0.52 | N.D. | N.D. | N.D. |
| Yangtianxuelv | 2034.07±52.93 | 405.39±5.67 | 773.13±15.14 | 249.38±7.36 | 11.73±0.21 | 15.52±0.54 | 42.26±1.32 | 0.18±0.03 | N.D. | N.D. |
| Yangxianxueya | 2512.71±78.85 | 689.32±21.48 | 1095.56±24.22 | 180.79±8.22 | 5.37±0.17 | 18.59±0.29 | 43.64±1.54 | 0.27±0.15 | 0.05±0.08 | 0.31±0.01 |
| Yashixiang | 1981.87±36.36 | 662.62±95.91 | 879.44±12.72 | 72.81±5.81 | 0.9±0.08 | 5.58±0.31 | 17.36±0.76 | N.D. | N.D. | N.D. |
| Yimengbiya | 1655.13±34.74 | 312.29±2.92 | 483.88±3.69 | 108.32±0.24 | 9.53±1.53 | 15.47±0.35 | 33.77±0.54 | N.D. | N.D. | N.D. |
| Yingdehongcha | 1767.29±58.92 | 319.27±8.36 | 424.6±9.56 | 146.96±1.77 | 3.57±0.05 | 10.88±0.3 | 26.06±0.24 | 0.02±0.01 | N.D. | N.D. |
| Yinghong9 | 1858.65±57.95 | 410.27±17.74 | 457.52±17.94 | 168.88±6.14 | 3.53±0.29 | 6.89±0.38 | 28.87±1.79 | 0.17±0.03 | N.D. | N.D. |
| Yingshanyunwu | 2046.27±150.5 | 410.29±39.79 | 1049.26±77.7 | 152.55±11.47 | 14.63±0.56 | 12.75±0.58 | 33.09±1.47 | 2.17±0.09 | N.D. | N.D. |
| Yongchunfoshou | 3145.97±43.44 | 1456.97±24.4 | 1442±25.25 | 331.26±6.94 | 1.03±0.05 | 8.76±0.4 | 14.22±0.22 | 2.23±0.03 | 0.38±0.15 | 0.38±0.01 |
| Yongxihuoqing | 1950.29±119.32 | 1759.67±290.34 | 936.97±54.52 | 116.34±11.52 | 0.21±0.02 | 5.27±1.84 | 9.36±0.63 | 0.14±0.05 | N.D. | N.D. |
| Yuhuacha | 1935.84±128.72 | 359.8±36 | 612.85±31.47 | 127.82±7.96 | 13.17±0.26 | 26.56±0.25 | 56.44±2.34 | 0.34±0.07 | N.D. | 0.16±0.02 |
| Zhenganbaicha | 1748.1±20.51 | 173.62±6.18 | 1751.48±14.48 | 95.82±2.98 | 15.5±0.29 | 12.53±0.13 | 38.84±1.47 | 0.07±0.04 | N.D. | N.D. |
| Zhenghebaimaohou | 1914.52±109.7 | 942.68±668.68 | 742.15±32.06 | 87.57±2.96 | 4.33±0.17 | 16.65±0.64 | 26.95±1.28 | 0.99±0.05 | N.D. | N.D. |
| Zhengshanxiaozhong | 1682.52±11.22 | 335.32±1.61 | 730.45±9.24 | 76.63±1.36 | 0.97±0.12 | 12.24±1 | 26.84±1.97 | N.D. | N.D. | N.D. |
| Zhufengshengcha | 2344.09±30.75 | 597.23±15.67 | 613.49±9.97 | 155.84±3.98 | 4.1±0.29 | 8.96±0.92 | 20.59±1.6 | 0.15±0.03 | N.D. | N.D. |
| Ziyangmaojian | 2400.51±68.59 | 778.61±202.74 | 709.06±15.89 | 182.08±3.72 | 9.5±0.54 | 25.65±1.06 | 51.14±2.44 | 0.19±0.09 | N.D. | 0.3±0.01 |
| Zunyihongcha | 1787.92±14.09 | 500.56±82 | 1153.94±5.9 | 126.42±1.44 | 1.53±0.05 | 6.28±0.32 | 28.63±1.63 | 0.31±0.05 | N.D. | N.D. |
| Zunyimaofeng | 1856.19±23.88 | 939.45±441.13 | 1169.62±16.99 | 51.85±2.98 | 4.43±0.09 | 13.7±0.47 | 22.2±0.88 | 0.05±0.03 | N.D. | N.D. |
